# NF-κB Drives the Synthesis of Melatonin in RAW 264.7 Macrophages by Inducing the Transcription of the Arylalkylamine-N-Acetyltransferase (AA-NAT) Gene

**DOI:** 10.1371/journal.pone.0052010

**Published:** 2012-12-21

**Authors:** Sandra Marcia Muxel, Marco Antonio Pires-Lapa, Alex Willian Arantes Monteiro, Erika Cecon, Eduardo Koji Tamura, Lucile Maria Floeter-Winter, Regina P. Markus

**Affiliations:** Department of Physiology, Institute of Bioscience, University of São Paulo, São Paulo, São Paulo, Brazil; University of Massachusetts Medical School, United States of America

## Abstract

We demonstrate that during inflammatory responses the nuclear factor kappa B (NF-κB) induces the synthesis of melatonin by macrophages and that macrophage-synthesized melatonin modulates the function of these professional phagocytes in an autocrine manner. Expression of a DsRed2 fluorescent reporter driven by regions of the *aa-nat* promoter, that encodes the key enzyme involved in melatonin synthesis (arylalkylamine-N-acetyltransferase), containing one or two upstream κB binding sites in RAW 264.7 macrophage cell lines was repressed when NF-κB activity was inhibited by blocking its nuclear translocation or its DNA binding activity or by silencing the transcription of the RelA or c-Rel NF-κB subunits. Therefore, transcription of *aa-nat* driven by NF-κB dimers containing RelA or c-Rel subunits mediates pathogen-associated molecular patterns (PAMPs) or pro-inflammatory cytokine-induced melatonin synthesis in macrophages. Furthermore, melatonin acts in an autocrine manner to potentiate macrophage phagocytic activity, whereas luzindole, a competitive antagonist of melatonin receptors, decreases macrophage phagocytic activity. The opposing functions of NF-κB in the modulation of AA-NAT expression in pinealocytes and macrophages may represent the key mechanism for the switch in the source of melatonin from the pineal gland to immune-competent cells during the development of an inflammatory response.

## Introduction

Melatonin (N-acetyl-5-methoxytryptamine) is synthesized through a highly conserved pathway involving the conversion of serotonin to N-acetylserotonin (NAS) by the enzyme arylalkylamine-N-acetyltransferase (AA-NAT) and the subsequent methylation of NAS by the enzyme acetylserotonin-methyltransferase (ASMT) [1].Besides the pineal gland and the retina, mononuclear and polymorphonuclear leukocytes also synthesize melatonin. In the last years we extensively demonstrated that the pineal gland is also a target for pathogen-associated molecular patterns (PAMPs), like LPS or pro-inflammatory cytokines, like TNF. These signals suppress nocturnal melatonin surge, facilitating the migration of leukocytes to the site of lesion [2]–[5]. In addition, LPS suppresses the synthesis of melatonin through activation the NF-κB pathway [6]–[8], while bacteria or phytohemaglutinine leads to the production of melatonin by macrophages and lymphocytes [9]–[11].

The NF-κB family is composed of five members of the Rel protein family: p50 (NF-κB 1), p52 (NF-κB 2), RelA (p65), RelB and c-Rel. These proteins form homodimers or heterodimers that are retained in the cytoplasm by inhibitory proteins that mask their nuclear localization sequence [12]. Upon activation by inflammatory mediators or PAMPs, NF-κB dimers translocate to the nucleus and bind to DNA-κB elements, resulting in transcriptional activation or repression. Although inflammatory stimuli can result in the triggering of a canonical NF-κB signaling pathway, changes in the composition of NF-κB dimers can alter the effects of the NF-κB pathway on gene regulation [13], [14]. Dimers with at least one subunit that contains the transactivation domains (RelA, RelB or c-Rel) usually induce gene transcription, whereas dimers that contain only p50 and p52 repress the expression of the target genes. Moreover, the interaction of NF-κB subunits with other modulators of gene transcription may alter the regulatory effect of a portion of the dimers. Therefore, the cellular context is highly relevant for NF-κB activity.

In this study, we characterized the role of the NF-κB pathway in the activation of the *aa-nat* promoter and the induction of melatonin synthesis in RAW 264.7 macrophages. We generated cell lines that express a red fluorescent reporter protein under the control of κB elements in the promoter and in the first intron of the *aa-nat* gene. Treatment of reporter cells with LPS induced both *aa-nat* transcription and melatonin synthesis, and these effects were blocked by pharmacological and siRNA-mediated inhibition of RelA and c-Rel NF-κB activity. In addition, we explored the function of macrophage-synthesized melatonin and showed that its interaction with melatonin G-protein-coupled receptor enhanced zymosan phagocytosis. Thus, our findings demonstrate for the first time that the bidirectional communication [15] between pineal and extra-pineal sources of melatonin is dependent on NF-κB nuclear translocation, which is a central event in the innate immune response, and that melatonin is part of the chemical arsenal that turns macrophages into professional phagocytes.

## Methods

### Melatonin Measurement

The melatonin contents of the supernatants from RAW 264.7 murine macrophages cells line [16] were measured through enzyme-linked immunosorbent assays (Melatonin ELISA, IBL, Hamburg, Germany) according to the manufacturer’s instructions. The cells (2×10^6^ in 300 µL) were stimulated with *Escherichia coli* LPS (500 ng/mL, 3 h; serotype 0127:B8; Sigma-Aldrich, St. Louis, MO) or *Saccharomyces cerevisiae* Zymosan A (zymosan; 20 µg/mL, 6 h; Sigma-Aldrich, St. Louis, MO). To inhibit the NF-κB pathway, the cells were incubated for 30 min with either the proteasome inhibitor acetyl-L-leucyl-L-leucyl-L-norleucinal (ALLN; 50 µM; Tocris, Minneapolis, MN) or pyrrolidinedithiocarbamate (PDTC; 25 µM; Tocris, Minneapolis, MN), which inhibits the binding of NF-κB dimers to κB DNA elements, before stimulation with LPS or zymosan.

### Cloning of *aa-nat* Promoter Region

The fragments derived from 5′ flanking regions of the *aa-nat* promoter (Genbank U77455.2) [17] were generated by PCR using total rat genomic DNA as a template. The aa-nat-kB-1 fragment was amplified using the primers F1 (5′-ccgctcgagctagagaccttcctgttctcctggtac-3′) and R1 (5′-cggaattccgaggaagctgtagcccgcattcccc-3′). The primers were designed to produce a fragment spanning from 349 bp upstream to 12 bp downstream of the start of the first exon. This fragment contains a single κB site (5′-agggggatt-3′) immediately upstream of the first exon (-45). The aa-nat-κB-2 fragment was amplified using the primers F1 (see above) and R2 (5′-cggaattcgttgggcaaatccctgggacttgctg-3′). Primer R2 is specific to a region upstream of the first intron (+487).Thus, the fragment aa-nat-κB-2 contains two κB binding sites (5′-agggggatt-3′ and 5′-gggatttgccc-3′), which bind to the last κB element of the promoter and the first in the intron. The aa-nat-*κ*B-1 and aa-nat-*κ*B-2 fragments were inserted into the pDsRed2-1 plasmid (Clontech, Mountain View, CA) using the *Eco*RI and *Xho*I restriction sites.

### Plasmids and Transfection

The pDsRed2-1-aa-nat-*κ*B-1, pDsRed2-1-aa-nat-κB-2 and pDsRed2-1 control plasmids were used to generate stably transfected macrophage cell lines. RAW 264.7 macrophages (2×10^6^) were seeded into 24-well plates for 24 hours. In sequence, macrophages were incubated for 72 h (37°C, 5% CO_2_) with a transfection reagent-plasmid complex. This complex was prepared by incubating for 20 min at room temperature, 3 µL of FugeneHD transfection reagent (Roche, Mannheim, Germany) diluted in 100 µL of serum-free 199 medium (Gibco BRL Products, Grand Island, NY) containing 2 µg of pDsRed2-1-aa-nat-κB-1, pDsRed2-1-aa-nat-κB-2 or pDsRed2-1 plasmid.

Stable expression of the exogenously introduced genes was achieved by culturing the transfected macrophages in the presence of neomycin sulfate (0.5 mg/mL; Gibco BRL products, São Paulo, Brazil). After 30 days of selective antibiotic pressure, the cloned macrophages were analyzed for DsRed2 expression using confocal microscopy.

### Transient Transfection of Small Interfering RNA (siRNA)

Transfected macrophages (1×10^5^ cells) were first plated on glass coverslips and maintained overnight. In sequence, the cells were incubated for 24 h with transfected reagents plus sense and antisense Turbofect-siRNA complexes diluted in 100 µL of RPMI 1640 medium (Gibco BRL Products, Grand Island, NY) containing 10% serum bovine. Turbofect-siRNA complexes were prepared by diluting 2 µL of Turbofect (Fermentas, Glen Burnie, MA) in 100 µL of serum-free RPMI 1640 medium (Gibco BRL Products, Grand Island, NY) containing 5 nM of one the following siRNA pairs:c-Rel sense (5′-ccgugcuccaaauaca-3′) and antisense (5′-ugcaguauuuggagcacgg-3′); RelA sense (5′-ccaucaacuaugaugaguudTdT-3′) and antisense (5′-aacucaucauaguugauggdTdG-3′); or scrambled-control siRNA sense (5′-ccuacgccaccaauuucgudTdT-3′) and antisense (5′acgaaauugguggcguaggdTdT-3′) (Bioneer, Alameda, CA).

### Stimulation of Transfected Macrophages with LPS and TNF

To assess the functional role of NF-κB binding sites in the *aa-nat* promoter, 2×10^5^ transfected macrophages containing either pDsRed2-1-aa-nat-κB-1 (aa-nat-κB-1), pDsRed2-1-aa-nat-κB2 (aa-nat-κB-2*)* or the control plasmid were plated on glass coverslips for 18 h and were incubated with LPS (30–1000 ng/mL) or mouse recombinant TNF (TNF; 30, 60 or 90 ng/mL; BD Pharmingen, São Paulo, Brazil) for 5–15 min. Unstimulated cells were used as a negative control. To inhibit the NF-κB pathway, the cells were pre-incubated for 30 min with 50 µM ALLN, 25 µM PDTC or vehicle alone. Later, the cells were stimulated with LPS (500 ng/mL for 10 min).

### Phagocytic Activity

To assess the ability of melatonin receptor ligands to modulate the phagocytic activity of RAW 264.7 macrophages, cells (2×10^5^) were plated on glass coverslips and incubated with melatonin (MEL; 1 nM; Sigma-Aldrich, St. Louis, MO) or luzindole (LUZ; 0.1, 0.3 or 1 µM; Tocris, Minneapolis, MN) for 30 min and subsequently incubated with fluorescent zymosan particles (1×10^5^; Sigma-Aldrich, St. Louis, MO) for 2 h.

### EMSA Assays

Nuclear protein extracts were obtained from RAW 264.7 macrophages (1×10^6^ cells) as previously described [18]. Subsequently, 8-µg samples of nuclear extracts from macrophages stimulated with LPS (500 ng/mL for 0, 2.5, 5 or 10 min) or with zymosan (10 µg/mL for 15 min) were incubated in binding buffer (10 mM Tris-HCl pH 7.5; 1 mM MgCl_2_; 50 mM NaCl; 0.5 mM DTT; 0.5 mM EDTA; 4% glycerol; and 0.02U poly-dIdC) for 20 min at room temperature. Next, 2 µg of an antibody against p50, RelA, p52, c-Rel, RelB or Bcl3 (Santa Cruz Biotechnology, Santa Cruz, CA) was added. After 45 min, the nuclear extracts were incubated with a ^32^P-labeled double-stranded NF-κB probe (5′-agttgaggggactttcccaggc-3′; Sinapse, São Paulo, Brazil) for 30 min at room temperature, and later the protein-DNA complexes were analyzed on a non-denaturing 6% polyacrylamide gel (Bio-Rad Laboratories, Richmond, CA) in TBE (Tris-Borate/EDTA) buffer. After drying, the gel was exposed to XAR-5 Kodak film (Rochester, NY, USA) for 72 h at −80°C and was developed.

### Detection of the AA-NAT Enzyme by Immunocytochemistry

LPS-stimulated RAW 264.7 macrophages (500 ng/mL for 1 h) were fixed with methanol/acetone (1∶1; 15 min at −20°C), permeabilized with 0.5% Tween 20 (30 min at 4°C) and incubated with rabbit anti-AA-NAT (S0689) or anti-phospho-AA-NAT (S0814) antibodies (24 h at 4°C; Sigma-Aldrich, St. Louis, MO). The macrophages were then incubated with Texas Red-conjugated anti-rabbit IgG antibody (ab6793) (1 h at 4°C; Abcam, Cambridge, MA). Nuclear DNA was stained with DAPI (15 min at 4°C; Invitrogen, Carlsbad, CA). The slides were analyzed by confocal microscopy (Zeiss LSM 510) using a 63× water-immersion objective. Texas Red was excited using a HeNe 543/633 laser and the emitted fluorescence was measured at 650 nm. DAPI was excited using a UV laser at 364 nm and the emitted fluorescence was measured at 435–485 nm. Random photographs were taken from three fields in each plate at a resolution of 2048×2048 pixels with identical configuration parameters (e.g., pinhole, scanning speed and laser strength). To quantify relative AA-NAT fluorescence intensity, we counted 30 cells in three different randomly chosen fields and analyzed the images using ImageJ 1.41 software (http://rsb.info.nih.gov/ij).

### Detection of DsRed2 and Zymosan Fluorescence

For immunofluorescence analysis, the cells were fixed in acetone/methanol (1∶1; 15 min at −20°C) and permeabilized with 0.5% Tween 20 (30 min at 4°C); nuclear DNA was stained with DAPI (300 µM; 15 min at 4°C). The slides were analyzed by confocal microscopy (Zeiss LSM 510) using a 63× water-immersion objective. DsRed2 was excited using a HeNe 543 laser and the emitted fluorescence was measured at 560 nm. Zymosan-conjugated Alexa Fluor 594 was excited using a HeNe 543/633 laser and the emitted fluorescence was measured at 560–615 nm. DAPI was excited using a UV laser at 364 nm and the emitted fluorescence was measured at 435–485 nm. Random photographs were taken from three fields in each plate at a resolution of 2048×2048 pixels with identical configuration parameters (e.g., pinhole, scanning speed and laser strength). To quantify DsRed2 fluorescence intensity, we counted 30 cells, in three different randomly chosen fields. Cells that had internalized three or more zymosan particles were counted as positive for phagocytosis. The results were normalized to the total number of cells per field.

### Statistical Analyses

The data are expressed as the means ± SEM. The groups were compared by one-way ANOVA variance analysis followed by the Newman-Keuls test. P values less than 0.05 were considered to be statistically significant. Straight lines were fitted using the minimal squares method and tested using analysis of variance. Inter-group variance was subdivided for testing the slope and the deviation from linearity.

## Results

### Melatonin Synthesis in Macrophages

**Figure 1:**
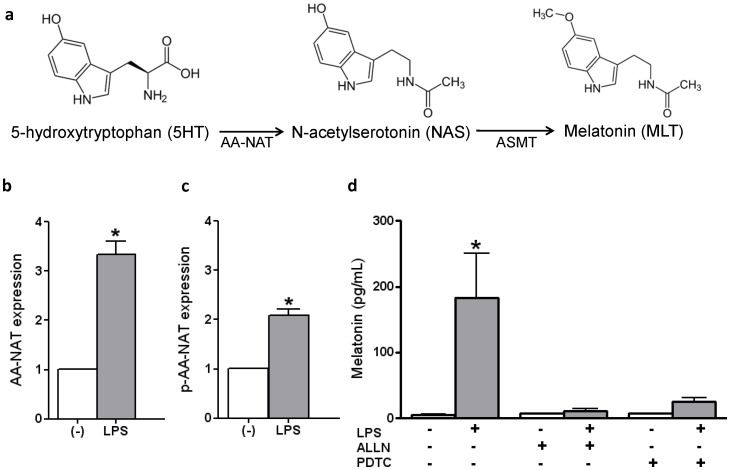
LPS-induced melatonin synthesis depends on the nuclear translocation of NF-κB in RAW 264.7 macrophages. (**a**) The conversion of serotonin (5-HT) to N-acetylserotonin (NAS) by the enzyme arylalkylamine-N-acetyltransferase (AA-NAT) is a key step in melatonin synthesis. This conversion is followed by N-acetyl-methyltransferase (ASMT)-mediated methylation of N-acetylserotonin. Expression of AA-NAT (b) and phospho-AA-NAT (p-AA-NAT)(c) detected by immunocytochemistry in RAW 264.7 cells, incubated (gray bars) or not (white bars) with LPS (500 ng/mL, 1 h). (d) LPS-induced melatonin synthesis by RAW 264.7 cells was blocked by incubation with ALLN (50 µM) or PDTC (25 µM) 30 min prior to stimulation with LPS. Each bar represents the mean ± SEM of values obtained in 3–5 independent experiments. *,p<0.05 compared to the respective control group.

LPS induced a significant increase in the expression of the native and phosphorylated forms of the enzyme AA-NAT in RAW 264.7 macrophages (Figure 1). This increase suggested that macrophages incubated with LPS produced melatonin, which was confirmed by measuring the presence of indolamine in the culture medium. LPS stimulation induced a 20-fold increase in melatonin concentration over the basal value (below 5 pg/mL) (Figure 1). This effect was mediated by the activation of the NF-κB pathway, as the inhibition of the nuclear translocation of NF-κB dimers by ALLN or the inhibition of NF-κB DNA binding by PDTC blocked the LPS-induced synthesis of melatonin (Figure 1).

#### Activation of the *aa-nat* promoter is regulated by κB elements

**Figure 2:**
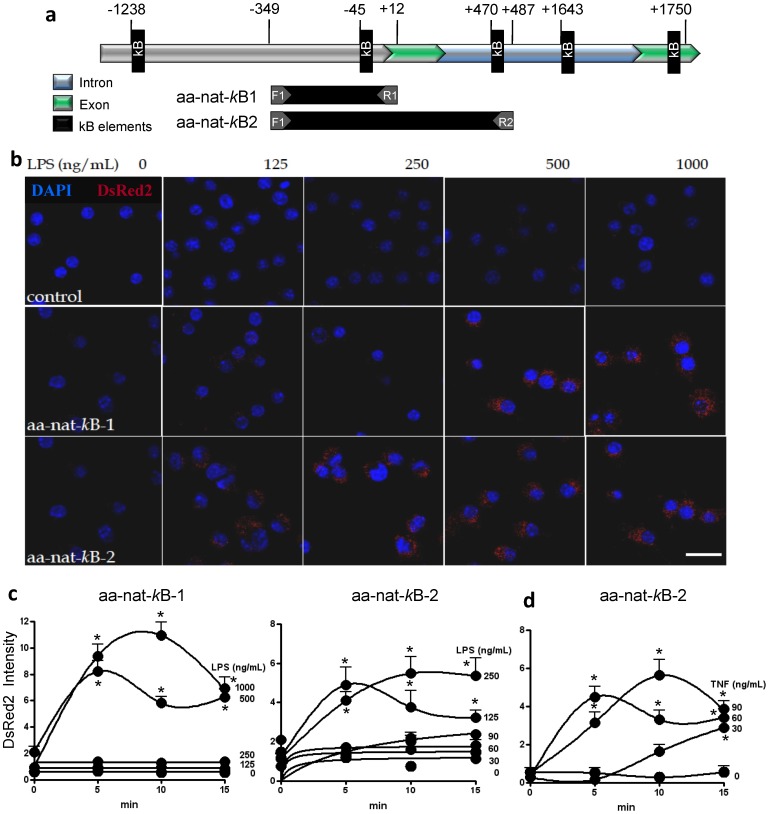
The dose-dependent activation of the *aa-nat*-promoter by LPS is driven by *κ*B elements. (a) A schematic representation of the 5′ upstream regulatory region of the rat *aa-nat* gene indicating the two putative *κ*B elements in the promoter region, two putative *κ*B elements in the first intron and the single κB element in the second exon (black rectangles). The regions included in the aa-nat-κB-1 and aa-nat-κB-2 constructs used in this study are indicated. The aa-nat-*κ*B-1 fragment spanned from 349 bp upstream into promoter to 12 bp downstream of the start of the first exon and contained one *κ*B site (5′-agggggatt-3′) upstream of the first exon (−349), and the aa-nat-*κ*B-2 fragment spanned from 349 bp upstream to 487 bp downstream of the start of the first exon and contained both the above *κ*B site and a second site (5′gggatttgccc 3′inside the first intron. The fragments were inserted into the pDsRed2-1 vector using *Eco*RI and *Xho*I restriction sites and the resulting constructs were used to transfect RAW 264.7 macrophages. (b) Confocal microscopy of RAW 264.7 cells transfected with pDsRed2-1-aa-nat-*κ*B-1, pDsRed2-1-aa-nat-*κ*B-2 or pDsRed2-1 control vectors following stimulation with 0, 125, 250, 500 or 1000 ng/mL LPS for 5 min. Red fluorescence demonstrates expression of the DsRed2 reporter protein. Blue fluorescence represents nuclei stained with DAPI. Scale bar = 20 µm. The images are representative of three different randomly chosen fields. The activation of the *aa-nat* promoter is demonstrated via the quantification of the DsRed2 absolute fluorescence intensities in RAW 264.7 cells transfected with pDsRed2-1-aa-nat-κB1 or pDsRed2-1-aa-nat-κB2 and stimulated with 0, 30, 60, 90, 125, 250, 500 or 1000 ng/mL LPS (c) or stimulated with 0, 30, 60 or 90 ng/mL TNF (d) for different times. The data shown represent the means ± SEM of DsRed2 fluorescence intensity of three independent experiments. For each data point, we counted 30 cells in three different randomly chosen fields.* p<0.05 from LPS zero at the same incubation time.

Based on *in silico* identification of putative κB elements in the *aa-nat* promoter and in the first intron [19], DNA fragments containing either one (aa-nat-κB-1) or two (aa-nat-κB-2) κB binding site sequences were generated by PCR. These fragments were later inserted into the pDsRed2-1 plasmid, which encodes the fluorescent DsRed2 reporter protein (Figure 2a). RAW 264.7 macrophages were transfected with the resulting plasmids to generate two macrophage reporter cell lines. Quantification of the fluorescence intensity of DsRed2-1 following LPS or TNF stimulation showed that both treatments activated the *aa-nat* promoter constructs in a dose-dependent manner (Figure. 2b–d). The increase in DsRed2 expression in aa-nat-κB-1 reached a maximal increase with 10 min LPS (1000 ng/mL), while in aa-nat-κB-2 the maximal increase was already obtained with 250 ng/mL of LPS. Importantly, both LPS and TNF are stimuli known to induce the nuclear translocation of NF-κB, providing further evidence that the NF-κB pathway triggers *aa-nat* transcription in macrophages.

**Figure 3:**
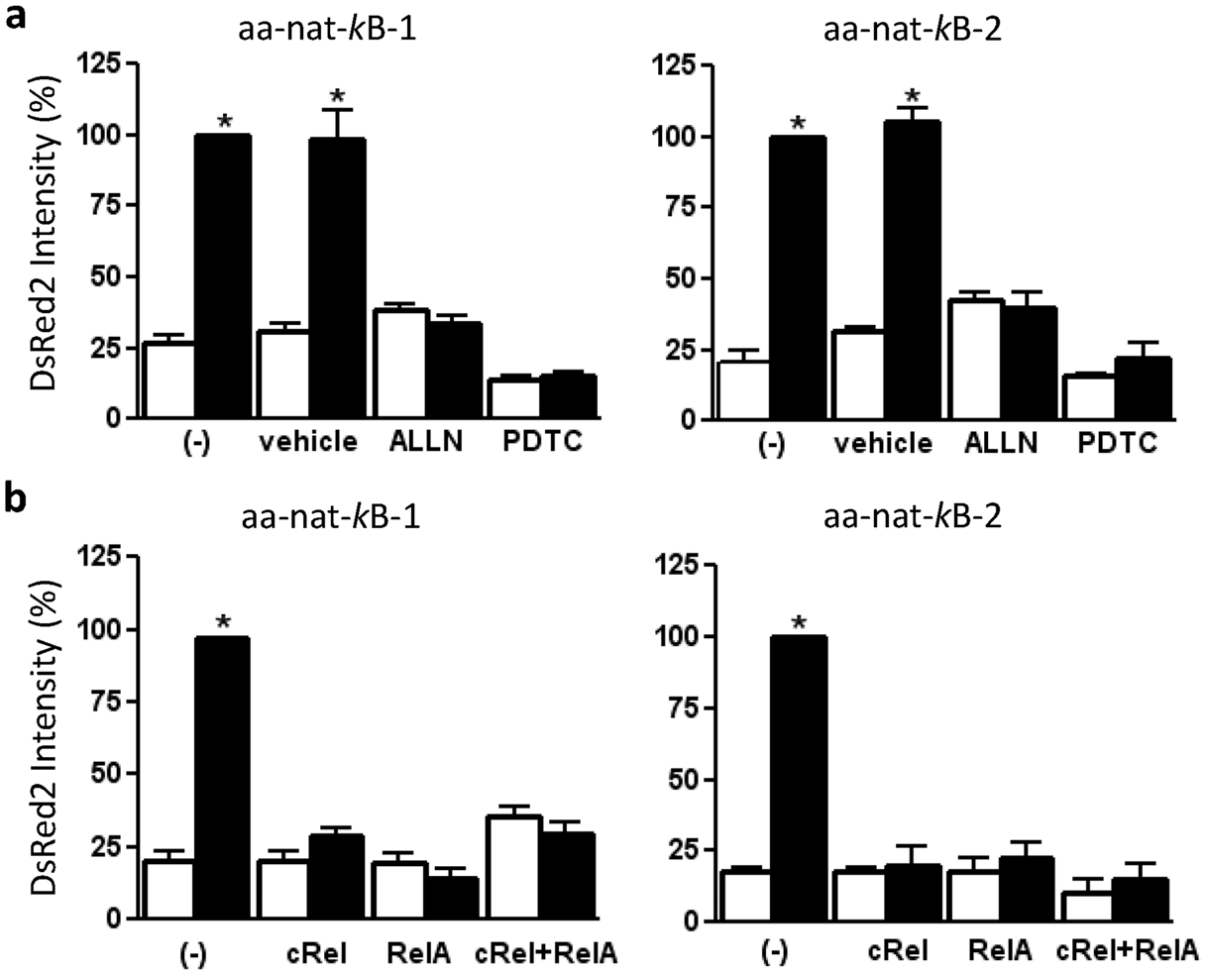
NF-κB-dependent activation of the *aa-nat* promoter. (a) The DsRed2 fluorescence intensity in ALLN- or PDTC-treated cells relative to that in LPS-stimulated cells (100%). The RAW 264.7 cells transfected with pDsRed2-1-aa-nat-κB-1 or pDsRed2-1-aa-nat-κB-2 were stimulated (black bar) or not (white bar) with LPS (500 ng/mL for 5 min) and in the presence or absence of the NF-κB inhibitors ALLN (50 µM) or PDTC (25 µM for 30 min). (b) The DsRed2 fluorescence intensity in siRNA-treated cells relative to that in LPS-stimulated cells (100%). The RAW 264.7 cells transiently transfected with siRNA (5 nM for 24 h) against the NF-κB subunits c-Rel or RelA or both (c-Rel+RelA); a scrambled siRNA (-) was used as a control. Each bar represents the mean ± SEM of the fluorescence intensity of DsRed2.For each data point, we counted 30 cells in three different randomly chosen fields. *, p<0.05 compared with control (white bars) (N = 3 independent experiments).

This hypothesis was further supported by experiments in which NF-κB activity was inhibited and the NF-κB proteins, RelA and c-Rel, were silenced by small interfering RNA. Both methods of inhibiting NF-κB activity blocked the transcription of the DsRed2 fluorescent reporter in the cells transfected with constructs containing either one (*aa-nat*-κB-1) or two (*aa-nat*-κB-2) κB binding site sequences (Figure 3). As both c-Rel and RelA are necessary for the induction of AA-NAT synthesis, we concluded that the c-Rel/RelA dimer is responsible for inducing melatonin synthesis in macrophages.

#### Melatonin enhances the phagocytic activity of macrophages through a paracrine mechanism

When the immune-pineal axis hypothesis was formulated, we speculated that the production of melatonin at the site of infection should facilitate the elimination of bacteria or other particles [19]. Thus, we next tested whether melatonin produced by macrophages could improve the phagocytic function of these cells by assessing the phagocytosis of zymosan.

**Figure 4:**
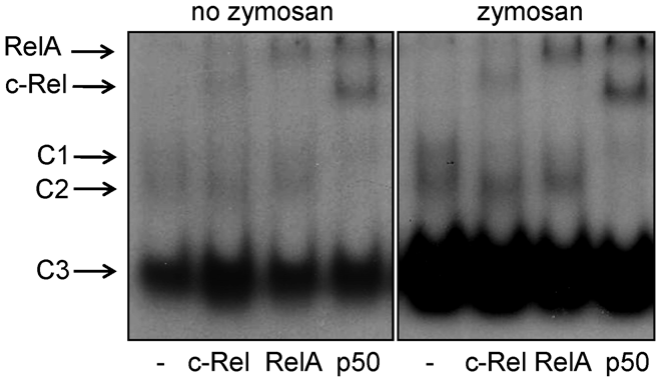
Zymosan induces the nuclear translocation of the NF-κB subunits cRel, RelA and p50 in RAW 264.7 macrophages. Super-shift assays with anti-cRel, anti-RelA and anti-p50 antibodies using nuclear extract protein (8 µg) from RAW 264.7 macrophages in the presence (10 µg/mL, 15 min) or absence of zymosan. C1-3 represents nuclear protein-NF-κB double-stranded oligonucleotide complexes.

**Figure 5:**
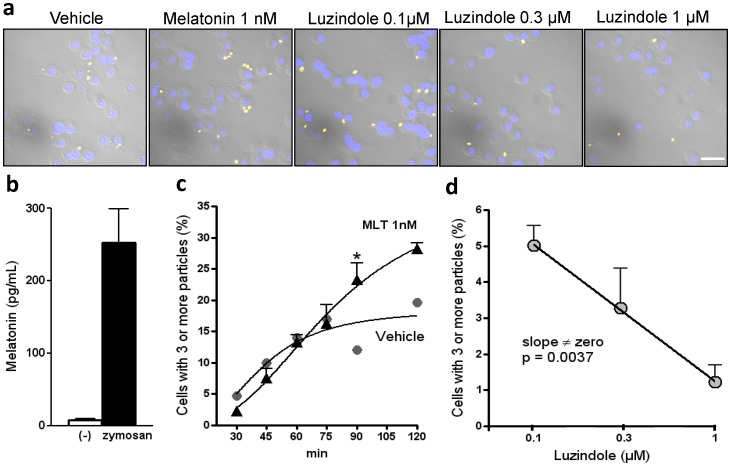
The inhibition of melatonin receptors reduces zymosan phagocytosis by RAW 264.7 macrophages. (a) Representative confocal microscopy images of RAW 264.7 cells (2×10^5^ cells) exposed to fluorescent zymosan (1×10^5^ particles) for 90 min. The cells were pre-incubated with melatonin (1 nM) or luzindole (0.1, 0.3 or 1 µM) for 30 min. Yellow fluorescence represents fluorescent zymosan. Blue fluorescence represents nuclei stained with DAPI. Scale bar = 20 µm. The images are representative of the three different randomly chosen fields. (b) Melatonin production in RAW 264.7 cells stimulated with (black bar) or without (−) zymosan (20 µg/mL for 6 h; N = 5−6). (c) Phagocytosis of fluorescent zymosan (1×10^5^ particles/2×10^5^ cells) by cells cultured with (black symbols) or without (gray symbols) melatonin (1 nM for 30 min). Each data point represents the mean percent ± SEM of cells with three or more phagocyted particles. For each data point, we counted 30 cells in 3 different randomly chosen fields. (d) The dose-response curve of luzindole inhibition of zymosan phagocytosis. The slope obtained by fitting the curve to a linear equation was significantly different from zero (p = 0.0037).

Super-shift assays showed that zymosan induced an increase in the three NF-κB-nuclear complexes observed in control macrophages (Figure 4). Antibodies selective for the c-Rel, RelA and p50 subunits induced a super shift of the C1 and C2 complexes. Although we cannot exclude the presence of RelA and c-Rel homodimers, the fact that C1 and C2 were completely super-shifted by p50 strongly indicates that zymosan induces the nuclear translocation of the p50/RelA and p50/c-Rel heterodimers. As expected, zymosan also induced the synthesis of melatonin (Figure 5).

To examine the function of macrophage-produced melatonin, we quantified the phagocytosis of zymosan in the presence of melatonin (1 nM) or luzindole (0.1–1 µM). The addition of melatonin to the culture medium increased the number of cells containing more than 3 zymosan particles, whereas luzindole, a competitive antagonist of melatonin receptors [20], reduced the number of cells containing 3 or more particles after 2 h of incubation in a dose-dependent manner (Figure 5). It is interesting to note that the concentration of melatonin produced by RAW 264.7 cells incubated with zymosan is around 1 nM and that the addition of 1 nM of exogenous melatonin double the number of cells that phagocyte three of more zymosan particles. This result strongly indicates that the NF-κB-induced synthesis of melatonin by macrophages serves to improve the macrophages phagocytic capacity.

## Discussion

This study demonstrates that the transcription factor NF-κB is able to induce AA-NAT expression in macrophages, resulting in the synthesis of melatonin. Moreover, macrophage-synthesized melatonin improves its phagocytic capacity. Regarding the pineal gland, the nuclear translocation of NF-κB, impairs noradrenaline-induced *aa-nat* transcription and melatonin synthesis [6], [7], [21], strongly suggesting that NF-κB activation has opposite effects in the pineal gland and in macrophages. Therefore, the cellular context and/or the specific NF-κB dimer translocation to the nuclei determine the effect of NF-κB activity on *aa-nat* transcription, providing a mechanism for switching the cellular source of melatonin.

In order to evaluate if *aa-nat* gene is regulated by NF-κB, we tested the expression of reporter constructs containing one or two κB binding sites in RAW 267.4 cells. The reporter of both constructs was transcribed upon stimulation of macrophages with LPS and, as expected, the reporter with two κB elements was expressed at higher levels than the reporter with one element. In addition, preventing NF-κB nuclear translocation or DNA binding inhibited the effect of LPS. Similar results were obtained when the NF-κB pathway was stimulated through TLR4 (LPS) or TLR2 and TLR6 (zymosan) [12], [22]. Therefore, in contrast to the inhibitory effect of NF-κB on melatonin production in pinealocytes [6], [7], the nuclear translocation of NF-κB induces melatonin synthesis in macrophages.

Previous reports have demonstrated that LPS induces the nuclear translocation of the p50/RelA, p50/c-Rel and c-Rel/RelA dimers [23]. All of these dimers contain a subunit with a transactivation domain known to activate gene transcription [12], [24]. Interestingly, in the pineal gland, both LPS and TNF induce the nuclear translocation of the p50/p50 dimer [6], [7], which is known to block gene transcription [12], [24]. Moreover, a diurnal rhythm of NF-κB nuclear translocation has been observed in pineal glands from normal rats. High levels of nuclear p50/p50 dimers are present during the day and a rapid reduction in the nuclear translocation of these dimers is observed as soon as the animals enter the dark phase [25].

An important difference between the activation of NF-κBin macrophages and pinealocytes is the nature of the NF-κB subunits. In pinealocytes stimulated with TNF or LPS, p50/p50 and p50/RelA are translocated to the nucleus [6], [7], while in macrophages, we also observed the nuclear translocation of c-Rel. Based on our super-shift assay results we suggest that the dimers c-Rel/c-Rel, c-Rel/p50 and c-Rel/RelA are translocated to the nuclei. Interestingly, in contrast to RelA, which is ubiquitously expressed and involved in many cellular functions, c-Rel is specific to active immune cells [26], [27]. In lymphocytes and macrophages, c-Rel homodimers and p50/c-Rel heterodimers are responsible for the LPS-induced expression of interleukin (IL)-2 [28], [29] and IL-12 [30], respectively. Another putative explanation for the opposing effects of NF-κB on *aa-nat* expression is the differential expression of co-activators and co-repressors in pinealocytes and macrophages [31]. In support of this idea, the *aa-nat* gene in rat pinealocytesis expressed only after the binding of CREB-P to the CRE regulating elements. This expression occurs *in vivo* upon stimulation of β-adrenoceptors [32]. Otherwise, in RAW 264.7 cells the activation of the NF-κB pathway *per se* result in the synthesis of melatonin. In summary, depending on the cellular milieu, activation of NF-κB could lead to inhibition or activation of the transcription of the mRNA that codifies AA-NAT, resulting in reducing or inducing melatonin synthesis. Disclosing this molecular mechanism is important for understanding how the switch between pineal and extra-pineal melatonin production of melatonin occurs when the immune-pineal axis is activated [18], [19], [32].

As the inflammatory or innate immune response is a coordinated organic response that has the purpose of combating aggression and restoring normal organism condition, the fact that the pivotal transcription factor switch the change in melatonin source depending on the cellular milieu strongly suggest that melatonin role changes from signaling darkness to being a mediator of this complex response [32]. Since the nineties it was shown that inhibition of the NF-κB pathway by melatonin reduces the transcription of genes and the expression of proteins involved in inflammatory responses [33]–[35], and more recently several studies show the pathophysiological relevance of this mechanism, as it participates either in the resolution of inflammatory responses [36]–[39], cell proliferation [40], and increases in macrophage phagocytic activity [41]. Therefore, the challenge of this study was to show that macrophage-synthesized melatonin could play a role in the ability of these cells to remove PAMPs from the extracellular milieu.

The local production of melatonin by activated immune cells may result in high local melatonin concentrations, due to the low volume of the intracellular milieu and the extracellular medium compared to the circulating blood. However, it was still necessary to prove that macrophage-secreted melatonin exerts a biological function. Phagocytosis of fungal particles leads to their destruction inside the phagolysosome. The recruitment of TLR to phagolysosomes is important for NF-κB nuclear translocation and the expression of cytokines and enzymes involved in the process of killing the foreign element. In this study, we show that zymosan induces the nuclear translocation of two NF-κB subunits that contain the transactivating domain: RelA and c-Rel. The nuclear translocation of these proteins results in the synthesis of melatonin by RAW 264.7 macrophages. In addition, exogenous melatonin at concentrations in the nanomolar range potentiates zymosan phagocytosis, whereas luzindole, a competitive antagonist of melatonin receptors, inhibits zymosan phagocytosis. Therefore, although a large body of evidence demonstrates that the effects of melatonin on immune-competent cells are linked to mechanisms independent of membrane receptors [42]–[45], in this study, we demonstrate that melatonin-induced potentiation of macrophage phagocytosis is mediated by membrane receptor activation.

In conclusion, the NF-κB pathway is the downstream mechanism that links the stimulation of macrophages by danger signals directly to the control of the key enzyme involved in melatonin synthesis. In turn, macrophage-synthesized melatonin enhances phagocytosis through autocrine action. In the context of the immune-pineal axis, the NF-κB pathway plays a dual role, depending on the cellular environment. Danger signals lead to an inhibition of melatonin synthesis in the pineal gland [6], [21], whereas they induce the synthesis of melatonin by macrophages. These apparently opposite effects are important for the initiation and resolution of the innate immune response, as a reduction in the nocturnal melatonin surge will allow leukocyte migration to sites of injury [2], [3], whereas the production of melatonin by immune-competent cells (independent of the circadian rhythm) is important for pathogen clearance.
